# Optic Neuritis in Chlorosis

**Published:** 1905-07-15

**Authors:** 


### OPTIC NEURITIS IN CHLOROSIS.

In several papers dealing with this subject, Dr. C. O. Hawthorne presents a number of clinical observations which he contends support the view that double optic neuritis occurring in chlorosis is dependent on thrombosis in the intracranial veins or venous sinuses. In one of the patients [1] there was, in addition to anæmia and double optic neuritis, paralysis of the left sixth cranial nerve, and this, it is urged, can hardly be explained as a result of a toxæmia—a condition which is sometimes advanced as the cause of the optic neuritis of chlorosis. Such an event suggests, on the contrary, a localised intracranial lesion, and as thrombosis is an admitted possibility in the circumstances, this is offered as the most probable explanation. The argument claims that the history of chlorosis includes instances of cerebral disturbances of all degrees of severity, ranging from coma and paralysis, often attended by double optic neuritis, and rapidly leading to a fatal issue, to instances of optic neuritis unattended by any other complication. In autopsies on fatal cases of the first group no lesion has been found other than intracranial thrombosis, and it must therefore be allowed that this is competent to cause optic neuritis. Hence when optic neuritis and an ocular paralysis occur in chlorosis, an intracranial thrombosis is a lesion which covers both events. Thus the conclusion is rendered probable that optic neuritis occurring alone has the same explanation. Support for this argument may be found in a case recorded by Dr. T. K. Monro, and recently summarised in The Hospital.[2] Here an anæmic girl developed right-sided convulsions followed by hemiplegia, paralysis of the sixth cranial nerves, and double optic neuritis. Thus the case may be presented as one intermediate between those instances in which severe cerebral disturbances lead to a fatal issue in chlorosis, and cases of the same disease in which double optic neuritis and a cranial nerve paralysis are the only unusual features. Double optic neuritis, in short, may be presented as the simplest example of those varieties of cerebral complications which occasionally occur in chlorosis, and which, in their most severe forms, lead to a fatal issue, attended, as shown by autopsy, by thrombosis in the intracranial sinuses and veins.

In another article by Dr. Hawthorne,[3] there are related a number of cases to show that various events, including double optic neuritis, which may at times be observed in anæmic patients, are best explained on the hypothesis of thrombosis. Two of the patients were the victims either of thrombosis or of embolism of the central artery of the retina, with consequent loss of sight in the affected eye. In neither patient was there a history of rheumatism or evidence of cardiac valve disease. Necessarily, therefore, it must be allowed that formation of blood-clot took place somewhere in the cardio-arterial system, and no cause could be found to explain this other than the anæmia. A third patient suddenly became blind in the right half of each visual field (right homonymous hemianopsia), and this condition was known to have existed without material change for a period of 15 years. There were no circumstances in the case to suggest a cerebral hæmorrhage, and the history, other evidence apart, renders tumour in the highest degree improbable. Again, therefore, thrombosis seems the most likely explanation, and the only discoverable condition likely to provoke this was the anæmia. In a fourth case the sole unusual feature in an otherwise ordinary case of chlorosis was the existence of double optic neuritis. From all these facts it is argued that as thrombosis is the almost inevitable explanation of retinal embolism and of hemianopsia when these events complicate chlorosis, it is reasonable to hold the same event responsible for optic neuritis when this occurs in similar circumstances. A rival interpretation able to cover all the cases in this series is certainly not easily provided.

[1] Lancet, April 30, 1904, and Polyclinic Jour., October 1904.
[2] The Hospital, July 8, 1905. [3] Practitioner, December 1904.

